# Real-time PCR using FRET technology for Old World cutaneous leishmaniasis species differentiation

**DOI:** 10.1186/s13071-016-1531-4

**Published:** 2016-05-03

**Authors:** Milli Nath-Chowdhury, Mugundhine Sangaralingam, Patrick Bastien, Christophe Ravel, Francine Pratlong, Juan Mendez, Michael Libman, Momar Ndao

**Affiliations:** National Reference Centre for Parasitology, Research Institute of the McGill University Health Centre, Montreal, QC Canada; Department of Parasitology-Mycology, Centre National de Référence des Leishmanioses, Centre Hospitalier Régional Universitaire of Montpellier and University Montpellier I (Faculty of Medicine), UMR CNRS 5290-IRD 224- UM1 et 2 “MIVEGEC”, Montpellier, France; Division of Experimental Therapeutics, Walter Reed Army Institute of Research, Silver Spring, MD USA; J.D. MacLean Centre for Tropical Diseases at McGill University, Montreal, QC Canada

**Keywords:** qPCR, Real-time PCR, *Leishmania*, Cutaneous, Diagnosis, FRET, Melting curve

## Abstract

**Background:**

Recently, there has been a re-emergence of cutaneous leishmaniasis in endemic countries and an increase in imported cases in non-endemic countries by travelers, workers, expatriates, immigrants, and military force personnel. Old World cutaneous leishmaniasis is caused primarily by *Leishmania major*, *L. tropica* and *L. aethiopica.* Despite their low sensitivity, diagnosis traditionally includes microscopic and histopathological examinations, and *in vitro* cultivation. Several conventional PCR techniques have been developed for species identification, which are time-consuming and labour-intensive. Real-time PCR using SYBR green dye, although provides rapid detection, may generate false positive signals. Therefore, a rapid and easy method such as a FRET-based real-time PCR would improve not only the turn-around time of diagnosing Old World cutaneous *Leishmania* species but will also increase its specificity and sensitivity.

**Methods:**

A FRET-based real-time PCR assay which amplifies the cathepsin L-like cysteine protease B gene encoding a major *Leishmania* antigen was developed to differentiate *L. major, L. tropica*, and *L. aethiopica* in one single step using one set of primers and probes. Assay performance was tested on cutaneous and visceral strains of *Leishmania* parasite cultures and isolates of other protozoan parasites as well as human biopsy specimen.

**Results:**

The assay readily differentiates between the three Old World cutaneous leishmaniasis species based on their melting curve characteristics. A single Tm at 55.2 ± 0.5 °C for *L. aethiopica* strains was distinguished from a single Tm at 57.4 ± 0.2 °C for *L. major* strains. A double curve with melting peaks at 66.6 ± 0.1 °C and 48.1 ± 0.5 °C or 55.8 ± 0.6 °C was observed for all *L. tropica* strains. The assay was further tested on biopsy specimens, which showed 100 % agreement with results obtained from isoenzyme electrophoresis and Sanger sequencing.

**Conclusion:**

Currently, there are no published data on real-time PCR using FRET technology to differentiate between Old World cutaneous *Leishmania* species. In summary, our assay based on specific hybridization addresses the limitations of previous PCR technology and provides a single step, reliable method of species identification and rapid diagnostic applications.

## Background

Leishmaniasis, an infection caused by obligate intramacrophage protozoa transmitted predominantly by the bite of an infected phlebotomine female sandfly is endemic throughout tropical and subtropical regions [1]. Cutaneous leishmaniasis (CL), which is characterised by ulcerative lesions on the skin (localised CL) and nonulcerative nodules (diffuse CL) is endemic in 88 countries with an estimated 1.5–2 million new cases every year and a total of 12 million cases worldwide [2, 3]. There has been a recent re-emergence of this disease in endemic countries and an increase in imported cases in non-endemic countries where travelers, workers, expatriates, immigrants, and military force personnel have been the main victims [2, 4–6]. CL is amongst the top ten diseases in tourists returning from tropical countries with skin problems [2, 7]. Old World CL (OWCL), prevalent in southern Europe, Mediterranean basin, Africa and the Middle-East is caused primarily by *Leishmania major*, *L. tropica* and *L. aethiopica* [4, 8], although CL cases due to *L. donovani* and *L. infantum* strains have also been reported [9, 10].

Diagnosis of CL traditionally includes microscopic examination of Giemsa-stained biopsy smears or tissue aspirates, histopathological examination, and *in vitro* cultivation. These methods however, in spite of their high specificity, are poorly sensitive and their sensitivity largely depends on the sampling procedure, parasite distribution and ad hoc expertise. Serological assays such as Enzyme-linked Immunosorbent Assay (ELISA), indirect fluorescence antibody test (IFAT) and western blot (WB) are preferred for the diagnosis of visceral leishmaniasis (VL) rather than CL due to the low titre of circulating antibodies against the parasite and cross-reactivity with other antigens (e.g. *Trypanosoma cruzi*) [11].

Molecular diagnostic methods such as PCR are the current preferred method of diagnosis due to their high specificity, sensitivity (98–100 %) and speed [12, 13]. Identification of the parasite at the species level is often crucial for epidemiological studies, transmission control measures, disease prognosis and choice of treatment [14, 15]. Before the advent of PCR protocols, the species of *Leishmania* was determined by enzyme-based assays such as Multi Locus Enzyme Typing (MLET) which were both time-consuming and labour-intensive. Several PCR-based techniques have been developed for species identification that require post-PCR processing such as electrophoretic analysis, PCR-restriction fragment length polymorphism (PCR-RFLP), PCR-ELISA and sequencing [16–19].

Real-time PCR not only allows the accurate detection and quantification of specific DNA in real time but also allows species identification without the requirement of post-PCR processing. Samples can be processed in less than one hour and the technique has been reported to rapidly differentiate single nucleotide mutations within a target DNA sequence [20]. To date, real-time PCR using SYBR green dye I has been reported several times for species discrimination [14, 21–24]. However, this method detects all amplified double-stranded DNA, including non-specific reaction products and can thereby generate false positive signals [25]. Recently, probe-based real-time PCR using Fluorescence Resonance Energy Transfer (FRET) identifies and distinguishes between New World tegumentary *Leishmania* species in clinical samples based on melting curve profiles with high specificity [26] thereby eliminating false positives.

Different PCR primers have been developed or applied for the detection and/or identification of *Leishmania* species [27]. One gene of interest, the *cpb* gene, encodes for cathepsin L-like cysteine proteinase B (*cpb*), a major antigen of *Leishmania* parasites and is conserved among the *Leishmania* species [28]. Its polymorphic and multi-copy nature presents an excellent opportunity for the development of species specific and sensitive primers [29]. Currently, separate primer sets targeting the *cpb* gene are needed to identify the OWCL species using conventional PCR, therefore requiring post-PCR processing [27] and this technique has demonstrated a lack of sensitivity in clinical samples [30].

In the present study, we describe a real-time PCR assay using FRET technology that is based on the amplification of the cathepsin L-like cysteine protease B (*cpb*) gene, to differentiate the main OWCL species: *L. major, L. tropica*, and *L. aethiopica*, in cultured parasite isolates and biopsy specimens. Currently, there are no published data on real-time PCR using FRET technology to differentiate between these OWCL species. This FRET-based real-time PCR assay requires specific hybridization between the probe and its target to generate a fluorescent signal thereby addressing the limitations of the SYBR green technology and providing a rapid, single step, reliable method of species identification for OWCL.

## Methods

### Ethics statement

Samples were obtained from multiple reference laboratories including the Canadian National Reference Centre for Parasitology/J.D. MacLean Centre for Tropical Diseases at McGill University (Montreal, QC), Centre Hospitalier Régional Universitaire of Montpellier and University Montpellier I (Montpellier, France) and Walter Reed Army Institute of Research (Silver Spring, Maryland) and were considered exempt. All samples used in this study were anonymized.

### *Leishmania* reference strain samples

DNA from *L. major*, *L. tropica*, and *L. aethiopica* promastigotes and cryopreserved promastigote cultures of various *Leishmania* reference strains were provided by the International Biological Resources Center for Leishmania, affiliated to the French National Reference Center for Leishmanioses, University Hospital Center of Montpellier, France. Additional DNA from *L. aethiopica* strains (promastigote stage) was provided by the Walter Reed Army Institute of Research, USA. An overview of all strains used is presented in Table 1.

**Table 1:** Overview of *Leishmania* strains used

Species	Strain	Parasite culture	Biopsy	Provider
*L. aethiopica*	MHOM/ET/96/WR2315^a^	No	No	WRAIR
	MHOM/SD/99/WR2885^a^	No	No	WRAIR
	MHOM/PH/2010/WR2970^a^	No	No	WRAIR
	MHOM/ET/83/130-83^c^	No	No	FNRCL
	MHOM/ET/90/DISKO^c^	No	No	FNRCL
	MHOM/ET/70/L96^c^	No	No	FNRCL
	MHOM/ET/81/1091-81^c^	No	No	FNRCL
	MPRV/ET/71/L111^c^	No	No	FNRCL
*L. tropica*	MHOM/IQ/65/L75	Yes	No	FNRCL
	MHOM/SU/74/K27	Yes	No	FNRCL
	I000/IL/98/LRC-L757	Yes	No	FNRCL
	MHOM/AF/06/NRCP2559^b^	Yes	Yes	NRCP
	MHOM/AF/05/NRCP358^b^	Yes	Yes	NRCP
	MHOM/SU/66/III^c^	No	No	FNRCL
	MHOM/KE/91/EB135^c^	No	No	FNRCL
	MHOM/MA/95/LEM3015^c^	No	No	FNRCL
	MHOM/IR/2000/LEM4036^c^	No	No	FNRCL
*L. major*	MHOM/IL/81/Friedlin	Yes	No	NRCP
	MHOM/DZ/05/NRCP684^b^	Yes	Yes	NRCP
	MHOM/BF/06/NRCP2082^b^	Yes	Yes	NRCP
	MHOM/BF/06/NRCP2204^b^	Yes	Yes	NRCP
	MHOM/TN/06/NRCP248^b^	Yes	Yes	NRCP
	MHOM/BZ/05/NRCP2620^b^	Yes	Yes	NRCP
	MRHO/SU/59/P-STRAIN^c^	No	No	FNRCL
	MHOM/SU/73/29-ASKH^c^	No	No	FNRCL
	MHOM/IL/83/IL24^c^	No	No	FNRCL
	MTAT/KE/00/T4^c^	No	No	FNRCL
	MHOM/DZ/89/LIPA228^c^	No	No	FNRCL
*L. chagasi*	MHOM/BR/74/M2682	Yes	No	FNRCL
*L. infantum*	MHOM/TN/80/IPT1	Yes	No	FNRCL
	MHOM/MA/67/ITMAP263	Yes	No	FNRCL
*L. donovani*	MHOM/IN/80/DD8	Yes	No	FNRCL
	MHOM/KE/55/LRC-L53	Yes	No	FNRCL
	MHOM/IQ/77/BUMM3	Yes	No	FNRCL
	MHOM/YE/86/LEM934	Yes	No	FNRCL
	MHOM/SD/90/2828	Yes	No	FNRCL
*L. mexicana*	MHOM/BZ/82/BEL21	Yes	No	FNRCL
	MNYC/BZ/62/M379	Yes	No	FNRCL
*L. panamensis*	MHOM/PA/71/LS94	Yes	No	FNRCL
*L. guyanensis*	MHOM/GF/79/LEM85	Yes	No	FNRCL
*L. peruviana*	MHOM/PE//84/UN56	Yes	No	FNRCL
	MHOM/PE/84/LC39	Yes	No	FNRCL
*L. braziliensis*	MHOM/PE/90/AC	Yes	No	FNRCL
	MHOM/CO/90/UA482	Yes	No	FNRCL
	MHOM/BR/75/M2904	Yes	No	FNRCL
	MHOM/BR/75/M2903b	Yes	No	FNRCL

*WRAIR* Walter Reed Army of Institute of Research, USA

*NRCP* National Reference Centre for Parasitology, Canada

*FNRCL* French National Reference Center for Leishmanioses, France

^a^DNA samples were provided by WRAIR

^b^DNA was extracted directly from patient biopsies

^c^DNA samples were provided by FNRCL

For all other strains, DNA was extracted directly from parasite

### Cutaneous lesion specimens

Cutaneous biopsy specimens which were sent to the National Reference Center for Parasitology between 2005 and 2006 for *Leishmania* testing, and were found to be positive in culture and by conventional PCR [31], were used to validate the real-time PCR assay. These biopsy culture isolates were also species typed by isoenzyme electrophoresis at the Walter Reed Army Institute of Research, USA. An overview of the specimens used is presented in Table 1.

### Cell culture and DNA extraction

Cryopreserved promastigotes and patient skin biopsies suspected of being positive for *Leishmania* were cultivated *in vitro* at 27 °C in RPMI 1640 medium (Wisent, St-Bruno, QC) supplemented with 20 % fetal bovine serum, non-essential amino acids (Wisent, St-Bruno, QC), MEM amino acids (Wisent), 1 mM sodium pyruvate, 2 mg/ml dextrose, 2 mM L-glutamine, 100 u/ml penicillin/streptomycin, and 25 mM HEPES. A set of control DNA standards from cultured promastigotes was prepared to determine the sensitivity of the real-time PCR. Promastigotes of *L. major*, *L. tropica* and *L. aethiopica* were suspended in PBS and uninfected human blood, counted in a Neubauer hemacytometer (Hausser Scientific, Horsham, PA) and diluted at a concentration of 10^6^ parasites/200 μl. Ten-fold dilutions were made to 10^−2^ parasites/200 μl. DNA was extracted from the promastigote dilutions and directly from patient skin biopsies using the QIAamp DNA Mini Kit (QIAGEN, Hilden, Germany) according to the manufacturer’s instructions. Following centrifugation and washing steps, DNA was eluted from the spin columns in 200 μl elution buffer and stored at -20 °C until use. Similarly, non-leishmanial protozoan DNA was extracted from blood specimen positive for *Plasmodium* species*, Trypanosoma cruzi* and *Trypanosoma brucei*, and from parasite cultures of *Toxoplasma gondii* RH strain (courtesy of Gary E. Ward, University of Vermont), *Giardia lamblia* ATCC® 30957 (courtesy of Gaetan Faubert, Institute of Parasitology, Quebec), *Cryptosporidium parvum* Iowa strain (courtesy of Michael Arrowood, Center for Disease Control) and *Entamoeba histolytica* ATCC® 30015.

### Primer and probe design

**Fig. 1:**
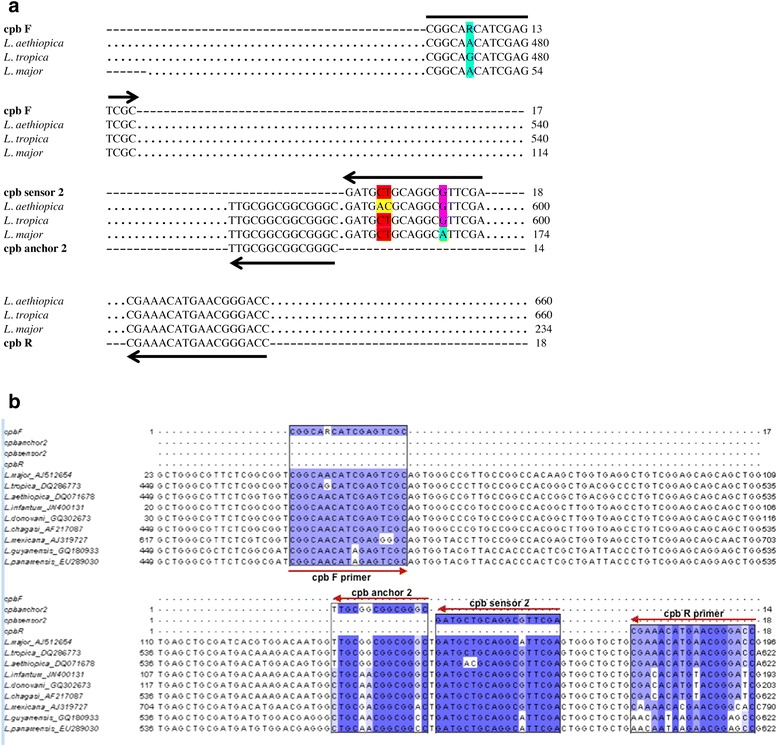
**a** Primer and fluorescence probe positions selected for FRET-based real-time PCR of *Leishmania* cathepsin L-like cysteine protease B gene. Sequences of forward (cpb F) and reverse (cpb R) primers were aligned with the corresponding target sequences. Forward primer harbors one wobble base (R = A/G). FRET hybridization probes (cpb sensor 2 and cpb anchor 2) were both designed antisense for detection of parasite. Sensor FRET hybridization probe was designed to be specific for cathepsin L-like cysteine protease B gene of *L. tropica* (GenBank Accession number: DQ286773) with one nucleotide mismatch difference from that of *L. major* (GenBank Accession number: AJ512654) and two nucleotide mismatch differences from that of *L. aethiopica* (GenBank Accession number: DQ071678). **b** Alignment of the OWCL species and species causing visceral and mucocutaneous leishmaniasis. Sequences are colour-coded by percentage identity

Consensus primers and probes, designed by TIBMol Biological (New Jersey, USA), were based on the alignment of *cpb* sequences for *L. major* (GenBank: AJ512654), *L. tropica* (GenBank: DQ286773) and *L. aethiopica* (GenBank: DQ071678). Alignment was done using ClustalW2 (v2.0.12, European Bioinformatics Institute, http://www.ebi.ac.uk). By comparing the *cpb* sequences of *L. major*, *L. tropica* and *L. aethiopica*, oligonucleotides were designed such that the FRET hybridization assay could selectively amplify DNA from each species, but allow for differences on melting curve temperature (Tm) analysis. The *cpb* sequence was almost identical for *L. tropica* and *L. aethiopica* with 96 % similarity. *L. major cpb* sequence shared 92 % similarity with that of *L. tropica* and 91 % similarity with that of *L. aethiopica*. A single primer and probe set was designed to amplify specimens from the OWCL species (Fig. 1a). Probes were designed to be specific for *L. tropica* and identify a single base-pair mismatch in *L. major* and two base-pair mismatches in *L. aethiopica*. The forward primer contains one wobble base to identify adenosine in the *cpb* gene of *L. aethiopica* and *L. major* as well as guanine in *L. tropica cpb* gene. Primers and probes (Table 2) were aligned with OWCL species and with those causing visceral and mucocutaneous forms (Fig. 1b).

**Table 2:** Primers and probes for the simultaneous detection and identification of OWCL species

	Name	Sequence (5’ ➔ 3’)	Function
Primers	cpb F	CGGCARCATCGAGTCGC	S
	cpb R	GGTCCCGTTCATGTTTCG	AS
Fluorescent-labeled probes	cpb sensor 2	TCGAACGCCTGCAGCATC—FL	AS
	cpb anchor 2	LC640-GCCCGCCGCCGCAA—PH	AS

*S* sense sequence, *AS* antisense sequence

### FRET-based real-time PCR

Real-time PCR reactions were performed using the Light Cycler Fast Start DNA Master HybProbe kit (Roche, Mannheim, Germany) and contained 2 μl of 10× Master Mix, 5 mM final MgCl_2_ concentration, 0.2 μM of each probe, 0.5 μM of each primer, and 2 μl of template DNA in a final volume of 20 μl. Real-time PCR cycling was performed on the Light Cycler 1.5 (Roche) with amplification at 95 °C for 10 min followed by 40 cycles of 95 °C for 5 s, 53 °C for 8 s, and 72 °C for 9 s, with single fluorescence acquisition at the end of each annealing step. Amplification was followed by a melting program of 95 °C for 20 s, 40 °C for 20 s, and a final increase to 85 °C at the rate of 0.2 °C/s with continuous fluorescence acquisition. To ensure the reproducibility of the assay, DNA from reference strains (*L. major* MHOM/IL/81/Friedlin, *L. tropica* MHOM/IQ/65/L75, and *L. aethiopica* MHOM/ET/96/WR2315) were included as positive controls in each run. An uninfected human DNA sample and a water sample were included as negative template and non-template controls respectively in each run.

### Direct sequencing and analysis

In order to ensure primer specificity and for validation, PCR products amplified from both promastigote and cutaneous biopsy DNA were subjected to agarose gel electrophoresis containing ethidium bromide and visualized under ultraviolet light. The band of interest was purified with QIAquick Gel Extraction Kit (QIAGEN, Hilden, Germany) and sent for direct Sanger sequencing to the McGill University and Génome Québec Innovation Centre, Montreal, Canada. Sequencing of samples was performed with cpb F and cpb R primers. The quality of the sequences were evaluated and edited with Geneious software (version 9.0.4 – restricted access) and the *Leishmania* species were identified by *BLASTm* accessible at *Genbank* (http://blast.ncbi.nlm.nih.gov/Blast.cgi). The sequences were subsequently aligned with the primers and probes using Jalview software (version 2.9.0b2 - restricted access).

## Results

### Species-specific FRET-based real-time PCR

**Fig. 2:**
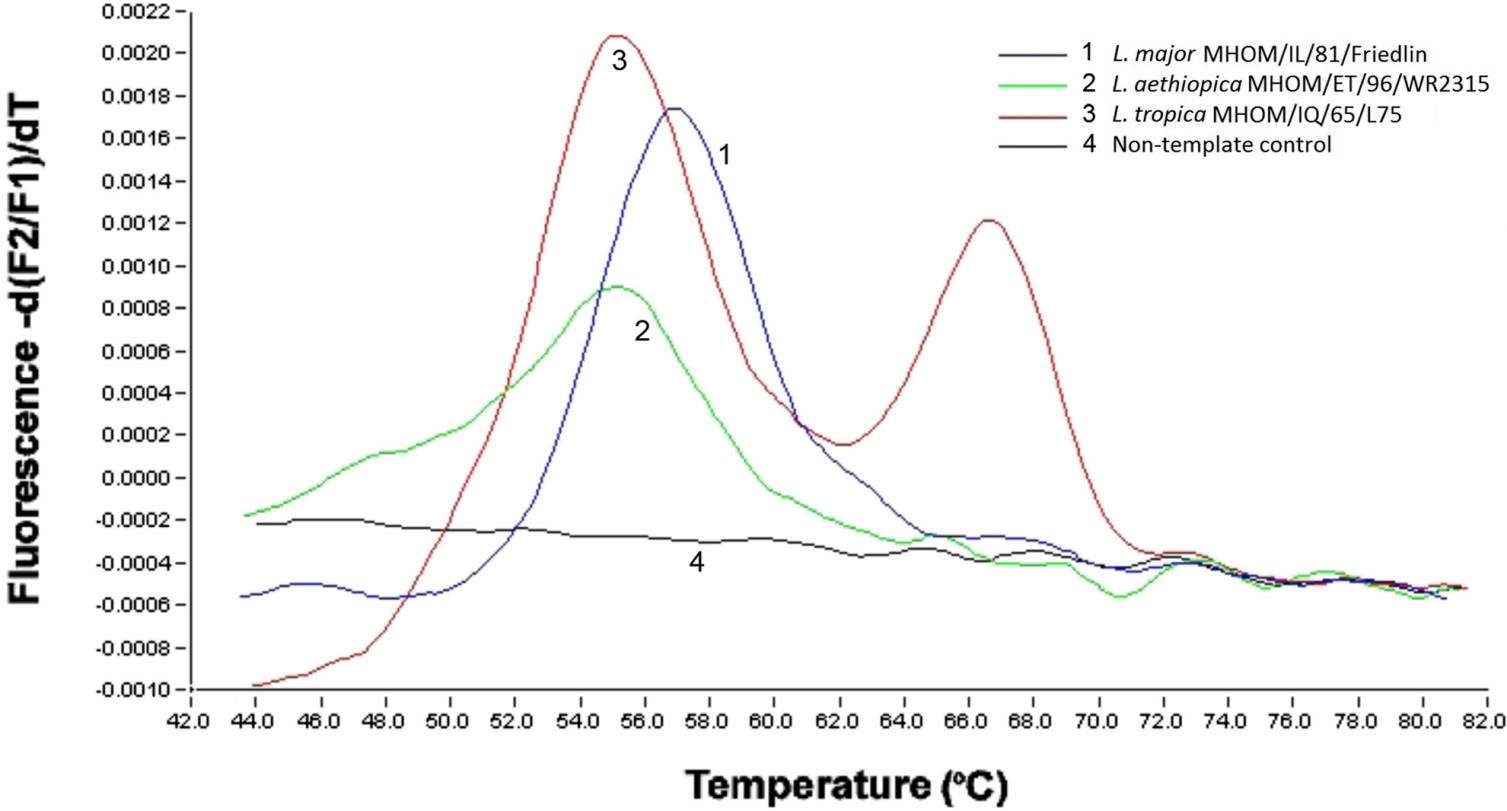
Example of a species-specific FRET-based real-time PCR result. OWCL species were differentiated by melting curve analysis. Single peaks visualized in *L. aethiopica* (Tm = 55.2 ± 0.5 °C) strains were distinguished from those of *L. major* (Tm = 57.4 ± 0.2 °C) or *L. tropica* with the latter showing double peaks (Tm = 66.6 ± 0.1 °C and 48.1 ± 0.5 °C or 55.8 ± 0.6 °C)

Using the FRET hybridization approach, species-specific real-time PCRs were performed on 28 DNA samples isolated from promastigote cultures of *L. tropica*, *L. aethiopica* and *L. major* reference strains. A species-specific amplicon with cpb F and cpb R primers was visualized in all *L. tropica*, *L. aethiopica*, and *L. major* strains. Melting curve analysis was able to differentiate between the species. A single Tm at 55.2 ± 0.5 °C for *L. aethiopica* strains was distinguished from a single Tm at 57.4 ± 0.2 °C for *L. major* strains. A double curve with a melting peak at 66.6 ± 0.1 °C and a peak at either 48.1 ± 0.5 °C or 55.8 ± 0.6 °C was observed for all *L. tropica* strains (Fig. 2).

The sensitivity of the real-time PCR was tested using serial dilutions of parasite DNA extracted from a known number of parasites. The *cpb* DNA of *L. major*, *L. tropica* and *L. aethiopica* could be detected at a level corresponding to 0.01 parasite per reaction volume of 20 μl. The detection limit was 10^−1^ parasite/200 μl of human blood, taking into account the amount of biological sample used in the reaction (2 μl of sample DNA) and the elution volume of the extracted DNA (200 ul).

### Non-species-specific real-time PCR

The specificity of the technique was validated using DNA from 18 strains of *Leishmania* causing mucocutaneous and visceral leishmaniasis from both the Old and New World as well as DNA from other protozoan parasites (Table 3). Non-specific low melting peak temperatures of 44.5 °C was observed for both *L. chagasi* and *L. infantum,* and 45.0 ± 0.7 °C for *L. donovani*. The primers cpb F and cpb R had no specificity for DNA from New World *Leishmania* species and non-leishmanial parasites (Table 3).

**Table 3:** Melting curve temperatures obtained from FRET-based real-time PCR assay

Species-specific			
Species	Strain	Peak	Tm (°C)
*Leishmania aethiopica*	MHOM/ET/96/WR2315	Single	54.5
	MHOM/SD/99/WR2885	Single	54.5
	MHOM/PH/2010/WR2970	Single	55.3
	MHOM/ET/83/130-83	Single	55.5
	MHOM/ET/90/DISKO	Single	55.5
	MHOM/ET/70/L96	Single	55.5
	MHOM/ET/81/1091-81	Single	55.5
	MPRV/ET/71/L111	Single	55.5
*L. tropica*	MHOM/IQ/65/L75	Double	55.3 | 66.8
	MHOM/SU/74/K27	Double	47.5 | 66.5
	I000/IL/98/LRC-L757	Double	55.5 | 66.8
	MHOM/AF/06/NRCP2559	Double	47.3 | 66.5
	MHOM/AF/05/NRCP358	Double	48.5 | 66.5
	MHOM/SU/66/III	Double	48.5 | 66.5
	MHOM/KE/91/EB135	Double	56.5 | 66.5
	MHOM/MA/95/LEM3015	Double	48.0 | 66.5
	MHOM/IR/2000/LEM4036	Double	48.5 | 66.5
*L. major*	MHOM/IL/81/Friedlin	Single	57.0
	MHOM/DZ/05/NRCP684	Single	57.3
	MHOM/BF/06/NRCP2082	Single	57.5
	MHOM/BF/06/NRCP2204	Single	57.3
	MHOM/TN/06/NRCP248	Single	57.3
	MHOM/BZ/05/NRCP2620	Single	57.3
	MRHO/SU/59/P-STRAIN	Single	57.3
	MHOM/SU/73/29-ASKH	Single	57.5
	MHOM/IL/83/IL24	Single	57.5
	MTAT/KE/00/T4	Single	57.5
	MHOM/DZ/89/LIPA228	Single	57.5
Non-species specific (causing visceral or mucocutaneous clinical manifestations)			
Species	Strain	Peak	Tm (°C)
*L. chagasi*	MHOM/BR/74/M2682	Non-specific single	44.5
*L. infantum*	MHOM/TN/80/IPT1	Non-specific single	44.5
	MHOM/MA/67/ITMAP263	Non-specific single	44.5
*L. donovani*	MHOM/IN/80/DD8	Non-specific single	45.8
	MHOM/KE/55/LRC-L53	Non-specific single	44.5
	MHOM/IQ/77/BUMM3	Non-specific single	44.5
	MHOM/YE/86/LEM934	Non-specific single	45.8
	MHOM/SD/90/2828	Non-specific single	44.5
*L. mexicana*	MHOM/BZ/82/BEL21	None	-
	MNYC/BZ/62/M379	None	-
*L. panamensis*	MHOM/PA/71/LS94	None	-
*L. guyanensis*	MHOM/GF/79/LEM85	None	-
*L. peruviana*	MHOM/PE/84/UN56	None	-
	MHOM/PE/84/LC39	None	-
*L. braziliensis*	MHOM/PE/90/AC	Undefined peak	-
	MHOM/CO/90/UA482	None	-
	MHOM/BR/75/M2904	None	-
	MHOM/BR/75/M2903b	None	-
Other protozoa			
Species	Strain	Peak	Tm (°C)
*Plasmodium falciparum*	10-4881	None	-
	10-4830	None	-
*Plasmodium malariae*	10-3066	None	-
*Plasmodium ovale*	10-2848	None	-
*Plasmodium vivax*	99-551	None	-
*Trypanosoma cruzi*	10-4342	None	-
	10-3447	None	-
	08-3341	None	-
	08-2636	None	-
	08-2634	None	-
*Trypanosoma brucei*	09-255	None	-
	08-3460	None	-
	00-659 (control)	None	-
*Toxoplasma gondii*	RH strain	None	-
*Entamoeba histolytica*	ATCC® 30015	None	-
*Cryptosporidium parvum*	Iowa strain	None	-
*Giardia lamblia*	ATCC® 30957	None	-

**Fig. 3:**
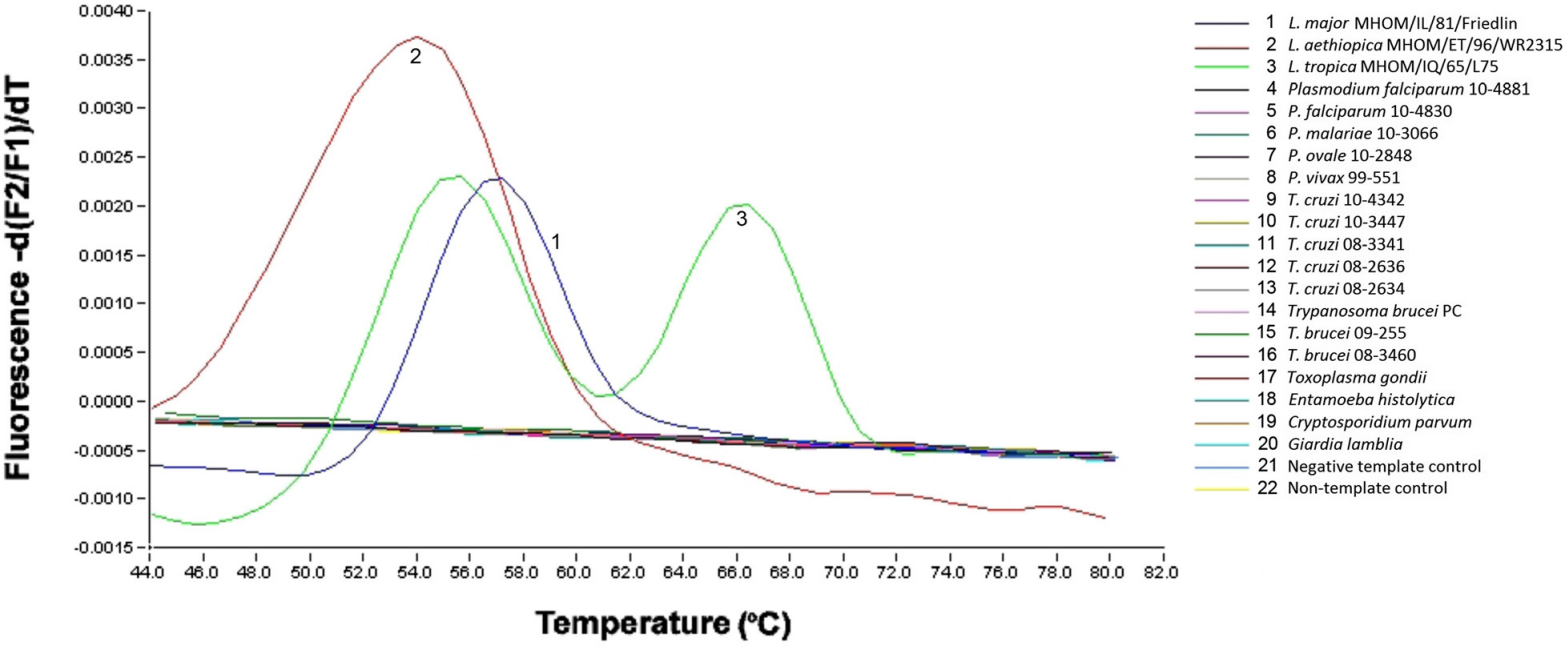
Cross-reactivity of OWCL FRET-based real-time PCR tested with other protozoa

We tested the cross-reactivity of our assay by analysing 17 samples known to contain other parasites including *Plasmodium falciparum*, *P. ovale*, *P. malariae*, *P. vivax*, *Trypanosoma cruzi*, *Trypanosoma brucei*, *Toxoplasma gondii*, *Entamoeba histolytica*, *Cryptosporidium parvum* and *Giardia lamblia* along with the reference strains *L. major* MHOM/IL/81/Friedlin, *L. aethiopica* MHOM/ET/96/WR2315 and *L. tropica* MHOM/KE/91/EB135. Final concentrations ranging from 0.08 μg/mL to 6.4 μg/mL were used to assess the specificity of the real-time PCR. Cross-reactivity was ruled out since amplification was only observed in the three reference strains (Fig. 3).

### FRET-based real-time PCR on biopsy samples

We used the FRET hybridization assay on DNA extracted from seven patients found positive for *Leishmania* by conventional PCR amplification of the 120 bp region of kinetoplast DNA [32]. By melting peak temperature comparison with the reference strains, five of these patients were identified as being infected with *L. major* and two with *L. tropica* (Table 1). Species identification for these samples was confirmed by isoenzyme analysis at the Walter Reed Army Institute of Research, USA and further confirmed by Sanger sequencing.

### Direct sequencing and analysis

A distinct band of 154 bp was visualized for all *L. major*, *L. tropica* and *L. aethiopica* strains. Similarly, a band was also seen at 154 bp for all species within the *L. (L.) donovani* complex.

Sequence comparison with available data in the *Genbank* database confirmed the amplification of the *Leishmania cpb* gene in all seven DNA biopsy specimen. Specimen positive for *L. major* by FRET real-time PCR showed 100 % identity with several *L. major cpb* gene sequences including those found in isolates from Tunisia (Accession No. JN400175) and those positive for *L. tropica* showed 99–100 % identity with several *L. tropica cpb* gene sequences deposited at the GenBank database.

## Discussion

We describe the development of a FRET-based real-time PCR using primers and probes targeting the *cpb* gene sequence to detect and identify the OWCL species (*L. major*, *L. tropica* and *L. aethiopica*) in a single step. This assay was able to produce unique, specific, and reproducible melting curves that could distinguish the three OWCL species (Fig. 2). The *cpb* sequence for each of the three species of interest has a single or double nucleotide difference from each other, allowing a difference of at least 2 °C between the Tm values, and enabling easy and reproducible distinction during melting curve analysis. The variation between strains within a single species is minor and did not interfere with the identification of any of the cutaneous strains tested. The Tms for all *L. major* and *L. aethiopica* strains showed standard deviations of only 0.2 and 0.5 °C, respectively.

*L. tropica* presented a unique bimodal peak with the second peak showing a consistent Tm. The first peak however showed greater Tm variation. It has been suggested that the first peak could indicate DNA fragments denaturing at lower temperatures such as primer-dimers and non-specific products which usually melt at a lower temperature than desired products [33]. However, all *L. tropica* strains tested showed only one discrete band on the agarose gel. Sequencing results from all *L. tropica* strains showed internal sequence differences. In the past, phylogenetic analysis and Multilocus Enzyme Electrophoresis (MLEE) have revealed genetic diversity and a high degree of allelic heterozygosity within *L. tropica* isolates [34, 35]. These differences may reflect distinct lineages of each strain and may explain the variation in melting characteristics of the first peak. Thus, species differentiation by melting curve analysis can be based on more than a single peak and its associated Tm [36–38]. In our assay, the unique bimodal peak from a single amplicon can be used in the detection and differentiation of *L. tropica* species from other cutaneous species.

Melting curve peaks for visceral species of *L. chagasi*, *L. infantum* and *L. donovani* were visible at 44.8 ± 0.6 °C. Peaks at low melting point temperatures are usually indicative of either primer-dimers due to their small size or amplification of non-specific products. However, gel results for these species indicate the presence of the target band at 154 bp. Although, the presence of a band confirms the identity of the parasite at the genus level, real-time PCR using melting curve analysis would enable species identification. In this case, the presence of a low Tm could be used as a possible marker to differentiate between *Leishmania* (*L.*) *donovani* complex and *L. tropica* complex. However, a prospective study would be necessary to assess and validate this.

The Tm values for the control reference DNA were highly reproducible on repeated melting curve runs. The melt peaks obtained with our technique are specific for *Leishmania* species and DNA from other protozoa was not amplified. Other investigators, using melting curve techniques such as high resolution melt analysis, found non-specific amplicons with non-leishmanial DNA although these did not overlap with the *Leishmania* species [39].

We also tested our FRET-based real-time PCR on seven patient biopsy samples received by the NRCP that were confirmed to be positive for CL by culture and conventional PCR, and were identified as *L. major* (five samples) and *L. tropica* (two samples) by the Walter Reed Army Institute of Research using isoenzyme electrophoresis. The five samples isoenzyme-typed as *L. major* showed Tm at 57.3 ± 0.1 °C and those typed as *L. tropica* showed double peaks at Tm 47.9 ± 0.9 and 66.5 °C, thus yielding Tms consistent with our previous results. This highlights the fact that our assay can be performed directly on patient samples without the need for isolation of parasites. We acknowledge that a small number of samples were used in this study. Ideally, this methodology could be further validated using a larger number of strains from a wide variety of sources and geographical areas. A future study conducted on a large sampling of clinical specimen would be necessary to validate this assay on patient DNA. Interestingly, one of the patients (MHOM/BZ/05/NRCP2620) positive for *L. major* in our real-time PCR assay as well in a blind panel for isoenzyme-typing by the Walter Reed Army Institute of Research, had demonstrated travel history to Belize. Given the above results, the presence of *L. major* in Belize comes as a surprise where this specie was not previously reported in this country. It is possible that the patient may have traveled to a *Leishmania major* endemic country prior to travel to Belize. However, rare cases of *L. major*-like strains have been reported and confirmed in New World countries [40, 41].

Species differentiation by real-time PCR is a highly effective tool in diagnostic laboratories that overcomes the drawbacks of conventional PCR. To date, most real-time PCR assays for cutaneous species differentiation have been developed using the SYBR Green method. Nevertheless, this method has failed to identify or differentiate between some species or requires separate PCRs for each species [14, 21, 23, 42, 43]. Recently, real-time PCRs have been developed in conjunction with FRET-based melting curve analysis for species and genus identification [44–46]. To our knowledge, this FRET-based nested real-time PCR was used to identify New World tegumentary leishmaniasis species targeting the mannose phosphate isomerase gene and the 6-phosphogluconate dehydrogenase gene by melting curve analysis [26]. Using this approach, results can be obtained within a short turnaround time and with relatively low costs.

Whole product melting curve analysis is recommended for species identification because it is more tolerant to small sequence differences that might result from intraspecific variation [47]. For diagnostic purposes, a probe-based real-time PCR assay is the preferred method due to its enhanced specificity as well as ease of analysis of the melting curves for species detection and differentiation. Melting curve analysis replaces post-PCR analysis involving nucleotide sequencing followed by comparison to a reference library of *Leishmania* strains [48].

Kinetoplast DNA, which has often been used as a target due to its high sensitivity in the diagnosis of leishmaniasis, can only identify the parasite to the genus or subgenus level [49]. The *cpb* gene, which has a relatively high copy number and showcases polymorphism between species, appear to give a more reliable species identification. It has been shown previously that the *cpb* gene could serve as an ideal target to differentiate between the different *Leishmania* species [29, 30, 45, 50] with a very high sensitivity (100 %) [51]. However, in contrast to previous assays targeting the *cpb* gene which were only able to distinguish between *Leishmania* complexes [52] or required multiple PCRs and post-PCR processing [15, 30, 45], here we developed unique primers that can differentiate between the three OWCL species in a single rapid step.

## Conclusion

In conclusion, we report a new highly sensitive FRET-based real-time PCR that can simultaneously identify the three species of Old World cutaneous leishmaniasis, *L. aethiopica*, *L. major* and *L. tropica,* from direct patient samples in a single step that could be used for rapid clinical diagnosis. However, as in any diagnostic procedure, the results of this assay need to be assessed in a routine diagnostic setting in light of the patient’s history and symptoms.

## Abbreviations

CL: cutaneous leishmaniasis
*cpb*: cysteine proteinase B
FRET: Fluorescence Resonance Energy Transfer
OWCL: Old World CL
Tm: melting curve temperature
